# Ergogenic effects of supplement combinations on endurance performance: a systematic review and meta-analysis of randomized controlled trials

**DOI:** 10.1080/15502783.2025.2524033

**Published:** 2025-07-06

**Authors:** Sebastian Zart, Michael Fröhlich

**Affiliations:** Department of Sports Science, RPTU University Kaiserslautern-Landau, Kaiserslautern, Germany

**Keywords:** Ergogenic aids, performance-enhancing substances, synergistic effects, performance, endurance

## Abstract

**Background:**

Supplements such as caffeine and sodium bicarbonate have been found to exert ergogenic effects on endurance performance. However, little is known about the effects of supplementary combinations on performance parameters. This review and meta-analysis aimed to summarize and analyze studies that investigated the effects of performance-enhancing supplements in combination and isolation on endurance (>35 s).

**Methods:**

A structured search was conducted in accordance with the PRISMA® statement and PICOS guidelines in the PubMed, Scopus and Dimensions databases in January 2024 without restriction to specific years. Sixteen studies that compared isolated and combined supplementation with placebo in an identical situation and tested their effects on endurance performance (time, distance, power) were included. The studies were all blinded, randomized controlled crossover studies that showed some concerns about risk of bias. Meta-analyses could be calculated for the supplement combinations caffeine (CAF) with sodium bicarbonate (SB) and CAF with beetroot juice (BJ), as sufficient studies were available for these two combinations. For the comparisons of the trials (e.g. placebo (PLA) vs. CAF; PLA vs. BJ; PLA vs. CAF-BJ; CAF-BJ vs. CAF; CAF-BJ vs. BJ), five studies each were included in the meta-analyses. A random-effects model and pooled standardized mean differences (SMD) according to Hedges’ g were used for the ergogenic effect.

**Results:**

The results showed no significant differences for either the isolated (CAF/PLA [CAF+SB studies]: SMD = 0.30, 95% CI [−0.12, 0.73], *p* = 0.16; SB/PLA: SMD = 0.31, 95% CI [−0.18, 0.80], *p* = 0.22; CAF/PLA [CAF+BJ studies]: SMD = 0.28, 95% CI [−0.08, 0.63], *p* = 0.13; BJ/PLA: SMD = 0.02, 95% CI [−0.33, 0.38], *p* = 0.90) or the combined supplement intake (CAF-SB/PLA: SMD = 0.43, 95% CI [−0.05, 0.91], *p* = 0.08; CAF-BJ/PLA: SMD = 0.33, 95% CI [−0.03, 0.69], *p* = 0.07) compared with the PLA trial. The subgroup analysis “test protocols” showed a borderline significance for cycling tests for the comparison of CAF-BJ with PLA (SMD = 0.39, 95% CI [−0.00, 0.78], *p* = 0.05). In addition, no significant differences were found between the isolated supplements and the co-ingestion (CAF-SB/CAF: SMD = 0.12, 95% CI [−0.30, 0.54], *p* = 0.57; CAF-SB/SB: SMD = 0.12, 95% CI [−0.29, 0.54], *p* = 0.56; CAF-BJ/CAF: SMD = 0.06, 95% CI [−0.30, 0.41], *p* = 0.76; CAF-BJ/BJ: SMD = 0.28, 95% CI [−0.08, 0.64], *p* = 0.13). According to the I^2^ statistics (0–22%), there was no or a low heterogeneity in the studies.

**Conclusions:**

In summary, it can be stated that the current state of research with few small studies and different methodological approaches (e.g. different types of sport, test protocols) only allows a limited reliable statement to be made about the combined effect of supplements. Although not statistically significant, trends observed in the forest plots suggest a potential advantage of combined supplement intake. Athletes should therefore test a possible benefit under their competition conditions. Further studies with a homogeneous design are required in the future in order to obtain more clarity about the tendencies of a combined ergogenic effect.

## 1. Introduction

Despite a very large number of supplements (e.g. phosphates, amino acids like tyrosine or polyphenol-rich foods like ginseng, green tea) that can be used to improve athletic performance [1], there is only clear scientific evidence for a few substances like caffeine or dietary nitrate [2]. According to the Australian Institute of Sport (AIS; https://www.ais.gov.au/), supplements are divided into four groups based on scientific evidence supporting their use. Group A includes performance supplements such as caffeine, beta-alanine, bicarbonate, nitrate, creatine and glycerol. There is strong scientific evidence for these supplements using evidence-based protocols [3–8]. In addition, studies on performance-enhancing effects are now available for the group of polyphenols [9]. Furthermore, according to AIS, the substances carnitine, curcumin, and menthol are classified in group B, as there is increasing scientific evidence for these substances [10–12]. The substances in groups C and D, on the other hand, either showed no benefit for athletes or no studies are currently available, or fall into the category of banned and heavily contaminated substances. Meta-analyses have already confirmed that the aforementioned dietary supplements from group A in particular can increase endurance performance. For this reason, the study examines the effectiveness of dietary supplements with existing but potentially limited evidence for endurance performance. An overview of the current state of research on the isolated effect of the supplements mentioned can be found in Table 1. Due to a lack of scientific evidence for the isolated effect of supplements from groups C and D, these were not included in the systematic review.

**Table 1.** Overview of reported ergogenic effects from meta-analyses in endurance performance.

Supplement	Study	Test/Exercise	Reported significant results
Beta-Alanine	Saunders et al. (2017) [5]	TT, TTE	ES = 0.18, 95% CI [0.08, 0.28], *p* < 0.05; σ^2^ = 72%
Caffeine	Christensen et al. (2017) [13]	TT between 45 s to 8 min	ES = 0.41, 95% CI [0.15, 0.68], *p* = 0.002; I^2^ = 0%
	Doherty & Smith (2004) [14]	TT, TTE	ES = 0.41, 95% CI [0.31, 0.51], *p* < 0.05; χ^2^(*df*, 75) = 45.9, *p* > 0.05
	Gomez-Bruton et al. (2021) [15]	total body impacts in team-sport athletes	SMD = 0.49, 95% CI [0.05, 0.93], *p* < 0.05; I^2^ = 49%, *p* = 0.117
	Gonçalves-Ribeiro et al. (2017) [16]	TT, TTE	TT: SMD = −0.40, 95% CI [−0.70, −0.11], *p* = 0.007; I^2^ = 0%, *p* = 0.87
	Grgic et al. (2020) [17]	Yo-Yo-Test Level 2	LDT: SMD = 0.17, 95% CI [0.02, 0.32], *p* = 0.022; I^2^ = 28%
	Grgic et al. (2020) [18]	Rowing TT	TT: SMD = 0.41, 95% CI [0.15, 0.68], *p* = 0.002; I^2^ = 0% MPO: SMD = 0.09, 95% CI [0.03, 0.15], *p* = 0.004; I^2^ = 0%
	Shen et al. (2019) [3]	TT (>5 min)	ES = 0.33, 95% CI [0.21, 0.45]; I^2^ = 0%
	Southward et al. (2018) [19]	TT (>5 min)	TT: SMD = 0.28, 95% CI [0.22, 0.61], *p* < 0.001; I^2^ = 63.7%, *p* < 0.001 MPO: SMD = 0.24, 95% CI [0.09, 0.39], *p* = 0.002; I^2^ = 0%, *p* = 0.989
Glycerol	Goulet et al. (2007) [8]	TTE	ES = 0.35, CI 95% [0.14, 0.56], *p* < 0.05; $x^{2}$(df, 3) = 7.82, *p* > 0.05
L-Arginine	Viribay et al. (2020) [20]	aerobic (≤VO_2_max) and anaerobic (>VO_2_max) performance tests	≤VO_2_max: SMD = 0.84, 95% CI [0.12, 1.56], *p* = 0.02; I^2^ = 89%, *p* < 0.001 >VO_2_max: SMD = 0.24, 95% CI [0.05, 0.43], *p* = 0.01; I^2^ = 0%, *p* = 0.85
Nitrate	d’Unienville et al. (2021) [6]	TT, TTE	2–5 min: SMD = 0.16, 95% CI [0.08, 0.23], *p* < 0.001; I^2^ = 0% 5–10 min: SMD = 0.18, 95% CI [0.09, 0.27], *p* < 0.001; I^2^ = 31% 10–30 min: SMD = 0.16, 95% CI [0.04, 0.28], *p* = 0.007; I^2^ = 41%
	Hoon et al. (2013) [21]	TT, TTE	TTE: ES = 0.79, 95% CI [0.23, 1.35], *p* = 0.006; I^2^ <25%
	Senefeld et al. (2020) [22]	TT, TTE	TT: SMD = 0.09, 95% CI [0.00, 0.017], *p* = 0.045 LDT: SMD = 0.32, 95% CI [0.14, 0.50], *p* = 0.001 FIT: SMD = 0.18 95% CI [0.04, 0.31], *p* = 0.013 TTE: SMD = 0.32, 95% CI [0.21, 0.44], *p* < 0.001
	Van de Walle & Vukovich (2018) [23]	TTE	TTE: SMD = 0.28, 95% CI [0.08, 0.47], *p* = 0.006; I^2^ = 0%
Polyphenols	d’Unienville et al. (2021) [6]	TT, TTE	10–30 min: SMD = 0.19, 95% CI [0.12, 0.26], *p* < 0.001; I^2^ = 0% 30–60 min: SMD = 0.12, 95% CI [0.03, 0.21], *p* = 0.008; I^2^ = 0%
	Somerville [9]		ES = 1.90, CI 95% [0.40, 3.39], *p* = 0.01; $I^{2}$ = 83%
Sodium bicarbonate	de Oliveira et al. (2022) [4]	TT, TTE	0.5–1.5 min: ES_0.5_ = 0.22, 95% CrI [0.13, 0.31], P(0.5–1.5 min > 1.5–5 min) = 0.915 1.5–5 min: ES_0.5_ = 0.14, 95% CrI [0.06, 0.21], P(1.5–5 min < 5–10 min) = 0.930 5–10 min: ES_0.5_ = 0.24, 95% CrI [0.12, 0.36], P(0.5–1.5 min < 5–10 min) = 0.622
	Christensen et al. (2017) [13]	TT between 45s to 8 min	SMD = 0.40 (95% CI [0.27, 0.54], *p* < 0.001
	Grgic et al. (2020) [17]	Yo-Yo-Test Level 2	LDT: SMD = 0.36, 95% CI [0.10, 0.63], *p* = 0.007; I^2^ = 14%
	Grgic & Mikulic (2022) [24]	Swimming TT (≈50 to 270 s)	>100 m: SMD = 0.22, 95% CI [0.10, 0.35], *p* < 0.001; I^2^ = 0%
	Lopes-Silva & Correia-Oliveira (2023) [25]	cycling TT	MPO: SMD = 0.42; 95% CI [0.21–0.63]; *p* = 0.001; I^2^ = 0% time: SMD = 0.22; 95% CI [0.02–0.43]; *p* = 0.03; I^2^ = 0%
	Matson & Tran (1993) [26]	TT, TTE	TT: ES = 0.32, 95% CI [0.00, 0.75] TTE: ES = 0.94, 95% CI [0.00, 2.87]
	Peart et al. (2012) [27]	exercise performance in different tests	≤2 min: ES = 0.45, 95% CI [0.05, 0.96] 2–10 min: ES = 0.34, 95% CI [0.06, 0.73]
	Saunders et al. (2022) [28]	exercise performance in different tests	ES = 0.37, 95% CI [−0.06, 0.92], P(increase > 0) = 0.962, P(increase > 0.2) = 0.784, P(increase > 0.5) = 0.263)

ES = Effect size; ES_0.5_ = Effect size based on the median value; FIT = Fatigue index tasks; LDT = Limited distance test; LTT = Limited time test; MPO = Mean power output; PPO = Peak power output; SMD = Standardized mean difference; TTE = Time-to-exhaustion; TT = time trial.

While the ergogenic effect of these supplements individually on athletic performance has been documented (see Table 1), knowledge of the effect of combinations of ergogenic supplements on athletic performance is limited. With the reviews by Ferrada-Contreras et al. [29] and Gilsanz et al. [30], only two studies are available that deal with the topic of co-ingestion of supplements.

Ferrada-Contreras et al. [29] examined the co-supplementation of beetroot juice with other supplements (caffeine, creatine, beta-alanine, citrulline, protein, amino acids, and carbohydrates). The combination of beetroot and other dietary supplements had no clear benefit in the studies examined with endurance tests. There were only positive effects for maximum strength or strength endurance. In addition, beetroot was not always administered in isolation, but as a ready-made mixed drink, so that no statements can be made about the isolated effect.

In the second review, only the combined effect of beta-alanine and sodium bicarbonate on the body’s buffer systems (reduction of H^+^ concentration) and athletic performance [30] was investigated. Five of the nine studies reviewed by Gilsanz et al. [30] indicated that co-supplementation improved performance to some extent. Owing to the underlying loads in these studies, co-supplementation was recommended for high-intensity exercises from 30 s to 10 min. Beyond this, we are not aware of any other review that has investigated the co-ingestion of supplements.

Therefore, the aim of the present study was to compile scientific findings from studies on co-supplementation, that examined ergogenic supplements in isolation and in combination during endurance exercise.

## 2. Methods

This systematic review and meta-analysis was conducted according to the PRISMA (Preferred Reporting Items for Systematic Review and Meta-Analyses) guidelines and the PICOS model (Population, Intervention, Comparison, Outcomes, Study Design) for defining the inclusion criteria [31,32].

### 2.1. Literature search strategies

A comprehensive literature search was carried out using the PubMed, Scopus and Dimensions databases. The search term basically linked the three keywords “supplements,” “combination” and “endurance” using the AND operator. For the placeholder “supplements,” all dietary supplements relevant to the review were linked using the OR operator. For the search with the keyword “combination,” alternative terms and synonyms were compiled and linked using the OR operator. The same procedure was used for the keyword “endurance.” Table 2 presents an overview of the search terms used in the three databases. Only studies in English that were listed in the aforementioned databases were considered for the review. There was no search for unpublished studies. In addition, the reference lists of the relevant studies were screened for further possible studies. The search was followed by a two-stage screening of studies. In the first step, titles and abstracts were checked for the inclusion criteria and excluded if they did not match. The second step involved full-text review. Here, too, all articles that did not meet the inclusion criteria were removed. Screening for suitable studies was performed independently by two authors (SZ and MF) and discrepancies were resolved by discussion between the two authors.

**Table 2.** Search terms used in the databases.

Databases	Search Strategy	Filters
PubMed	((caffeine) OR (sodium bicarbonate) OR (nitrate) OR (beetroot) OR (L-citrulline) OR (L-arginine) OR (beta-alanine) OR (polyphenols) OR (blackcurrant) OR (menthol) OR (carnitine)) AND ((combin*) OR ((coingestion) OR (co-ingestion))) AND ((performance-enhancing) OR (ergogenic) OR (enhancement) OR (increase) OR (improve) OR (athletic performance)) AND ((physical endurance) OR (endurance))	Randomized Control Trials Humans
Scopus	(TITLE-ABS-KEY (caffeine OR {sodium bicarbonate} OR nitrate OR beetroot OR {L-citrulline} OR {L-arginine} OR alanine OR polyphenols OR blackcurrant OR menthol OR carnitine) AND TITLE-ABS-KEY (combin* OR coingestion OR {co-ingestion}) AND TITLE-ABS-KEY ({performance-enhancing} OR ergogenic OR enhancement OR increase OR improve OR {athletic performance}) AND TITLE-ABS-KEY (endurance OR {aerobic exercise} OR {anaerobic exercise}))	Controlled Clinical Trial or Controlled Study Human
Dimensions	(caffeine OR “sodium bicarbonate” OR nitrate OR beetroot OR “L-citrulline” OR “L-arginine” OR alanine OR polyphenols OR blackcurrant OR menthol OR carnitine) AND (combined OR combination OR (coingestion OR “co-ingestion”)) AND (“performance-enhancing” OR ergogenic OR enhancement OR increase OR improve OR athletic performance) AND (physical endurance OR endurance)	title and abstract

### 2.2. Inclusion and exclusion criteria

The following inclusion criteria were defined for the literature search according to the PICOS model. The study population consisted of healthy male and female participants of all ages. A person is considered a healthy subject if no injuries or acute or chronic illnesses were present or known at the time of the study and the person was able to fully access their individual performance capacity within the scope of a study. The intervention investigated the combined effect of two relevant supplements (beta-alanine, caffeine, carnitine, L-arginine, L-citrulline, menthol, nitrates such as beetroot, polyphenols such as New Zealand blackcurrant, and sodium bicarbonate) on endurance performance (exercise >35 s) in a time trial (e.g. competition distance) or time-to-exhaustion test [33]. In studies in which a test was performed repeatedly, the individual load of the intermittent test protocol had to be at least 35 s long. The selection of the supplements was based on the available meta-analytical findings and the classification of the supplements by the AIS. L-arginine and l-citrulline were included in the search as both substances, like nitrates in the body, increase the nitrogen content (NO) in the tissue [34]. Carnitine was also used for the search term, as this substance could be effective during exercise lasting more than 30 minutes [35]. On the other hand, creatine and curcumin were not included in the search because their primary effect is on anaerobic exercise lasting less than 30 s and an improved ability to regenerate [11,36,37]. For comparison, the differences in placebo (PLA) versus supplement 1 alone, supplement 2 alone and a combination of both supplements were analyzed using a crossover design. Consequently, each subject completed all conditions and the results on ergogenic effects are based on within-subject comparisons. All performance-related variables such as time, distance or power from time trials or time-to-exhaustion tests were considered outcomes. Finally, the included studies had to be at least single-blind randomized placebo-controlled trials.

Studies conducted with animals or sick or injured subjects, or in which more than two supplements were administered (e.g. pre-work-out boosters) and the effects could not be clearly assigned, were excluded. We also excluded studies in which the supplement combinations were not additionally measured in isolation and in which there was no real control group taking a substance that represented PLA. In addition, studies were excluded if the full text was not available, blinding was not applied, or they were review articles, case reports or commentaries.

### 2.3. Data extraction

The following information was extracted from the selected studies: study source (authors and year of publication), supplementation (type of supplement, duration of intake, dosage, time of intake before exercise), subject characteristics (sample size, sex, personal details such as age, body weight, sports discipline), type of exercise used to test an ergogenic effect, and results (any measurement that could be used to assess physical performance).

In five studies [38–42], performance-related values for the four conditions were not fully reported in the text but were instead presented in figures. Therefore, as a first step, the authors were contacted via e-mail to request the raw data. Since no raw values were provided, the WebPlot Digitizer tool [43] was used to extract the measured values from the diagrams. The WebPlot Digitizer tool can guarantee almost perfectly valid and reliable data extraction [44]. We compared the reported values with the read-out values on the basis of five studies, and there was an average deviation of 0.58. Consequently, the small deviation should not affect the results.

### 2.4. Quality and risk of bias assessment

The quality of each included study was assessed using the Cochrane risk-of-bias tool for crossover trials (version 2), as this tool was specifically developed for studies in crossover design and can therefore also assess biases related to period and carryover effects. Quality assessment of the articles was performed independently by two authors (SZ and MF). The assessment was based on the categories “bias arising from the randomization process,” “bias due to deviations from intended interventions,” “bias due to missing outcome data,” “bias in measurement of the outcome,” and “bias in selection of the reported result.” The risk of bias was rated as low, with some concerns, or high. To determine the agreement between raters, Cohen’s kappa (κ) was calculated and the proposed Landis and Koch [45] rating was applied, with κ < 0: poor agreement; 0 < κ ≤ 0.2: slight agreement; 0.2 < κ ≤ 0.4: fair agreement; 0.4 < κ ≤ 0.6: moderate agreement; 0.6 < κ ≤ 0.8: substantial agreement; 0.8 < k ≤ 1.0: almost perfect agreement.

### 2.5. Meta-analysis

To support this systematic review, meta-analytical calculations were performed if a sufficient number of studies were available for a supplementary combination. This was the case for the supplement combinations caffeine (CAF) with sodium bicarbonate (SB) as well as CAF with beetroot juice (BJ). If several results were available, the most significant outcome (mean ± standard deviation) was included to measure performance. Random-effects meta-analyses were performed using the SPSS 29 software (IBM Corp., Armonk, NY, USA) using the inverse variance model and restricted maximum likelihood method. A random-effects model was chosen because heterogeneous intervention effects can be assumed due to different settings in the primary studies (e.g. test type, population, supplementation). The effects were reported using the standardized mean difference (SMD) according to Hedges’ g (corrected) and their 95% confidence intervals (CI), which were calculated using the means, standard deviations, and case numbers of individual studies. To interpret the SMD, Cohen’s thresholds for trivial (< 0.2), small (0.2–0.5), moderate (0.5–0.8), and large effect sizes (> 0.8) were applied. The a priori significance level for all comparisons was set at *p* < 0.05. The Q-test was used to determine heterogeneity in SMD. The I^2^ statistic was calculated as an indicator of the percentage of total variation observed within studies due to true heterogeneity rather than the chance, to avoid error when using the Q statistic to assess heterogeneity. The I^2^ values ranged from 0 to 100%, with a low proportion of inconsistency between 25% and 50%, a medium proportion of heterogeneity between 50% and 75%, and a high proportion of heterogeneity with an I^2^ value > 75%. To estimate the possible range in which the effect sizes of future studies are likely to fall, 95% prediction intervals were calculated for the pooled random effect sizes [46]. Sensitivity and subgroup analyses (test type: time trial vs. time-to-exhaustion; athletes: elite vs. sub-elite; test protocol: cycling vs. other activities) were performed in the presence of heterogeneity. Egger’s regression test was used to check for asymmetry in the funnel plots. In addition, publication bias was tested using the trim-and-fill method.

## 3. Results

### 3.1. Study selection

**Figure 1.**
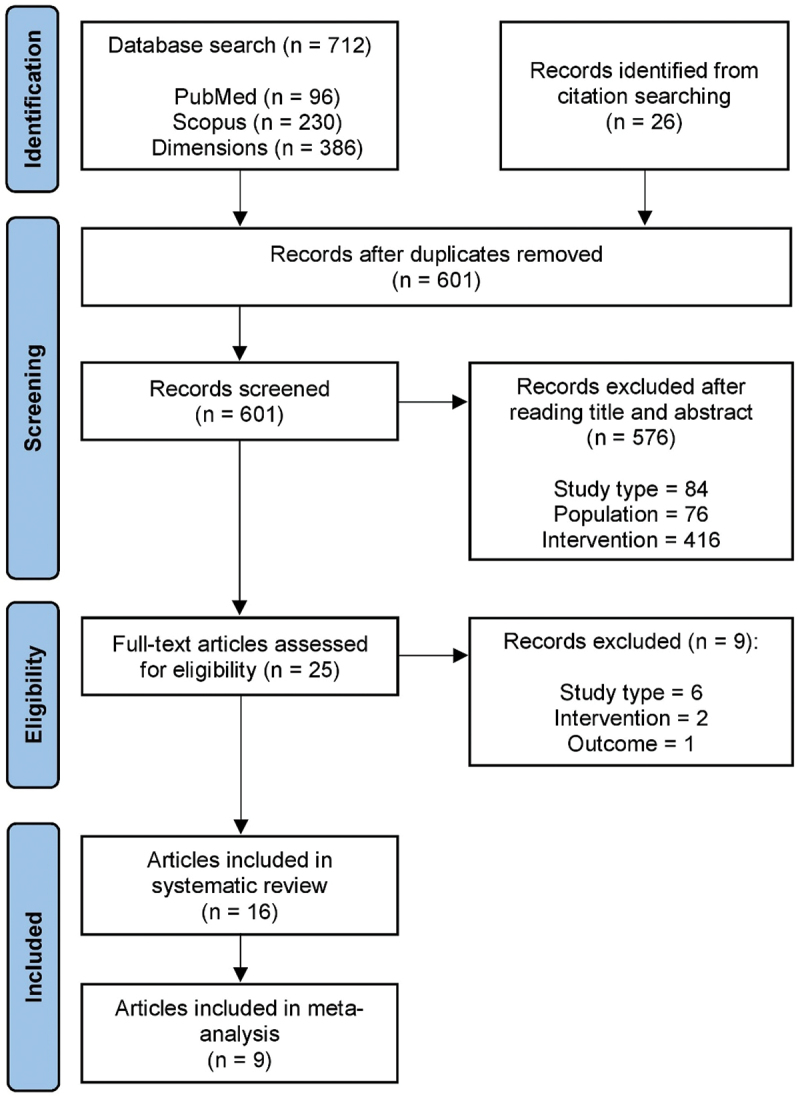
Flowchart of literature search according to PRISMA guidelines.

The database search identified 712 records. Additional identification of 26 potential articles was made possible by screening the references. After removing 137 duplicate articles, 601 articles were screened based on their titles and abstracts. The screening process excluded 576 articles from analysis. Most studies did not examine supplements in isolation and in combination. Consequently, the inclusion criteria were checked for 25 full text articles. Finally, 16 articles met all inclusion criteria (Figure 1).

### 3.2. Quality and risk of bias assessment

**Figure 2.**
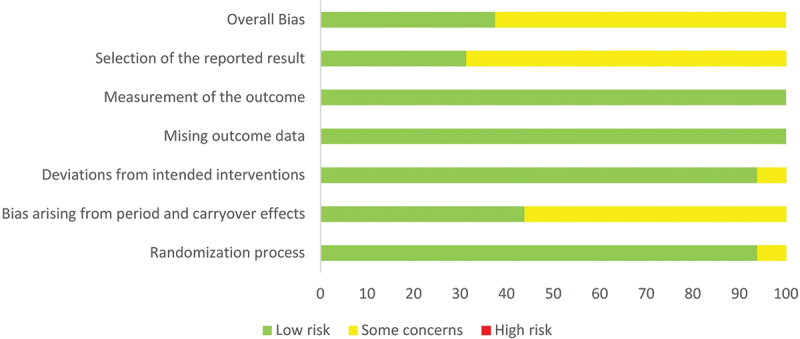
Risk-of-bias graph expressed as percentages.

**Figure 3.**
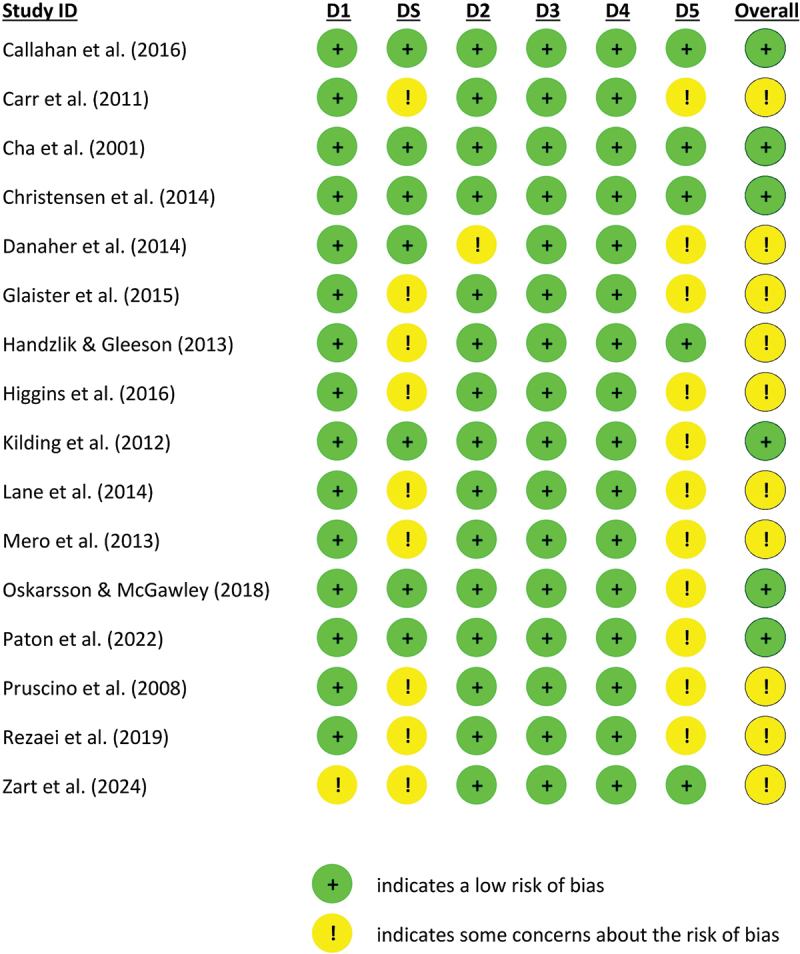
Summary of the risk of bias. D1: randomization process; D2: bias arising from period and carryover effects; D3: deviations from the intended interventions; D4: missing outcome data; D5: measurement of the outcome; D6: selection of the reported result.

The percentage distribution of the risk of bias for the dimensions is shown in Figure 2, and a detailed assessment of the dimensions for each study is shown in Figure 3. The inter-rater reliability for the different dimensions of the Cochrane risk of bias tool was between κ = −0.09–1.0 and thus showed a poor to perfect agreement. According to the statistics, there was no agreement for the dimension “bias due to deviations from intended interventions” (κ = −0.09), although the assessments were identical between the reviewers in 81,25% of cases (Kappa paradox due to imbalance in case distribution). All other dimensions had a κ > 0.3, with a perfect match for the dimensions “bias due to missing outcome data” and “bias in measurement of the outcome.”

### 3.3. Study characteristics

In total, all studies included 166 subjects with a range of 2–24 (Table 3). Half of the studies had sample sizes less than 10 participants [38,39,47–52]. Ten studies examined only male subjects [38,39,41,42,47,50,52–55] and one study examined only female subjects [40]. In four studies, both sexes were represented [48,49,56,57], and no further information on sex was reported in one study [51]. The participants included elite [40,42,47,50,51,56,57], sub-elite [41,48,49,54,55] and recreational athletes [38,39,52,53]. In these studies, tests were conducted to examine the performance-enhancing effects of supplements in the sports of running [49], cycling [38–41,47,52–55,57], rowing [48,56], swimming [42,50], and karate [51]. In 13 studies, CAF were combined with SB [48,50,51,53,54,56], carnitine [38], BJ [40,41,49,57] or blackcurrant [52,55]. Other combinations included SB with beta-alanine [39,42] and beetroot crystals [47].

**Table 3.** Characteristics of the crossover studies included in the systematic review.

**Study**		**NEA**	**Dosage;** **Time before test**	**Duration** **(days)**	**Sample**	**Exercise** **Protocol**	**Main Outcomes (M ± SD; Median (IQR); %)**
Callahan et al. (2017) [47]		BRC	15 g/day (5 mmol ${NO}_{3}^{-}$); days before the test	3	n = 8 well-trained male cyclists (age: 34 ± 7 yr; body mass: 73.8 ± 10.1 kg; VO_2_max: 65.2 ± 4.2 ml/kg/min; BMI: 22.3 ± 2.0 kg/m²	4 km TT	↔ cycling time (p > 0.05) ↔ Mean power (p > 0.05) BRC-SB: 336.10 ± 17.28 s; 393 ± 54 W BRC-PLA: 337.41 ± 17.11 s; 388 ± 54 W PLA-SB: 335.78 ± 16.94 s; 394 ± 52 W PLA-PLA: 338.10 ± 18.04 s; 386 ± 55 W
			15 g; 60 min	1			
		SB	0.3 g/kg; from 150 min a part of the dose every 15 min	1			
Carr et al. (2011) [48]		CAF	6 mg/kg; 30 min	1	n = 8 well-trained rowers 6 men (body mass: 82.2 ± 12.2 kg) 2 women (77.5 ± 6.4 kg)	2 km TT	↔ rowing time (likelihood = 74%) ↑ Mean power (likelihood > 75%) CAF-SB: 402.6 ± 21.6 s; 352 ± 63 W CAF-PLA: 400.8 ± 22.5 s; 354 ± 67 W* PLA-SB: 404.4 ± 23.4 s; 348 ± 67 W PLA-PLA: 403.8 ± 23.4 s; 346 ± 61 W
		SB	0.3 g/kg; 90 min	1			
Cha et al. (2001) [38]		CAF	5 mg/kg; 60 min	1	n = 5 healthy, male rugby players (age: 19.4 ± 0.36 yr; height: 178.9 ± 1.74 cm; body weight: 74.3 ± 1.59 kg; body fat: 19.2 ± 0.36 %: VO_2_max: 53.2 ± 1.59 mL/kg/min)	TTE at 80% VO_2_max after preload at 60% VO_2_max	↑ cycling time CAF-CAR: 32.75 ± 21.22 min^#^ CAF-PLA: 20.29 ± 19.57 min PLA-CAR: 24.06 ± 16.14 min*^+^ PLA-PLA: 14.35 ± 10.69 min
		CAR	15 g; 60 min	1			
Christensen et al. (2014) [56]		CAF	3 mg/kg; 45 min	1	n = 12 elite rowers 6 male open-weight (age: 25 ± 1 yr; body weight: 92 ± 3 kg)5 male light-weight (age: 24 ± 3 yr; body weight: 75 ± 3 kg)1 female light-weight rower (27 yr, 63 kg)	6 min TT	↑ total distance (p < 0.05) ↑ mean power (p < 0.05) CAF-SB: 1877 ± 97 m*^#^; 400 ± 58W*^#^ CAF-PLA: 1878 ± 97 m*^+^; 400 ± 58 W*^+^ PLA-SB: 1860 ± 96 m^+#^; 389 ± 57 W PLA-PLA: 1865 ± 104 m; 393 ± 61 W
		SB	0.3 g/kg; 75 min	1			
Danaher et al. (2014) [39]		BA	4.8 g/day; days before test	28	n = 8 healthy, recreationally active males (age: 26.2 ± 1.9 yr; body mass: 79.8 ± 2.11 kg; height: 179.0 ± 2.2 cm; VO_2_peak: 51.0 ± 2.5 ml/kg/min)	TTE at 110 % Wmax	↑ cycling time (p < 0.01) BA-SB: 150 ± 32.33 s (16%)* BA-PLA: 147 ± 37.22 s (14%)* PLA-SB: 132 ± 38.13 s (2%)PLA-PLA: 129 ± 30.94 s (0%)
			6.4 g/day; days before the test	14			
		SB	0.3 g/kg; 90 min	1			
Glaister et al. (2015) [40]	BJ		70 mL (7.3 mmol ${NO}_{3}^{-}$); 150 min	1	14 competitive female cyclists (age: 31 ± 7 yr; body mass: 61.6 ± 6.0 kg; height: 1.69 ± 0.07 m)	20 km TT	↑ cycling time (p = 0.001) ↑ mean power (p = 0.001) BJ-CAF: 34.76 ± 1.31 min; 203 ± 22 W BJ-PLA: 35.33 ± 1.50 min; 194 ± 22 W PLA-CAF: 34.62 ± 1.26 min*^+^; 205 ± 21 W*^+^ PLA-PLA: 35.37 ± 1.70 min; 194 ± 25 W
	CAF		5 mg/kg; 60 min	1			
Handzlik et al. (2013) [41]	BJ		70 mL (4 mmol ${NO}_{3}^{-}$); 150 min	1	14 healthy well-trained males (age: 22 ± 3 yr; body mass: 76 ± 7 kg; height: 177 ± 1 cm; VO_2_max: 63 ± 10 mL/kg/min)	TTE at 80% VO_2_max after preload at 60% VO_2_max	↔ cycling time (p > 0.096) BJ-CAF: 1463 ± 774 s (46%) BJ-PLA: 1238 ± 1002 s (23%) PLA-CAF: 1153 ± 564 s (15%) PLA-PLA: 1003 ± 480 s (0%)
			70 mL (4 mmol ${NO}_{3}^{-}$); 75 min	1			
	CAF		5 mg/kg; 75 min	1			
Higgins et al. (2016) [53]	CAF		5 mg/kg; 60 min	1	13 healthy, noncycling trained males (age: 21 ± 3 yr; body mass: 76 ± 12 kg; height: 178 ± 6 cm; peak power output: 230 ± 34 W; VO_2_max: 46 ± 8 mL/kg/min)	TTE at 100% peak power output	↑ cycling time (p < 0.05) CAF-SB: 367 s (333–402)^#^ CAF-PLA: 399 s (350–415)^+^ PLA-SB: 313 s (284–448) PLA-PLA: 358 s (290–433)
	SB		0.3 g/kg; 60 min	1			
Kilding et al. (2012) [54]	CAF		3 mg/kg; 60 min	1	10 well-trained cyclists (age: 24.2 ± 5.4 yr; body mass: 79.1 ± 7.2 kg; height: 179.0 ± 5.1 cm)	3 km TT	↑ cycling time (p < 0.05) ↑ mean power (p < 0.05) CAF-SB: 226.0 ± 10.5 s*; 382 ± 39 W* CAF-PLA: 226.5 ± 9.4 s*; 381 ± 37 W* PLA-SB: 225.9 ± 11.3 s*; 383 ± 44 W* PLA-PLA: 228.7 ± 10.8 s; 373 ± 41 W
	SB		0.3 g/kg; serially between 120 and 90 min	1			
Lane et al. (2014) [57]	BJ		140 mL (8.4 mmol ${NO}_{3}^{-}$); 8-12 hrs	1	24 competitive cyclists 12 male (age: 31 ± 7 yr; body mass: 73.4 ± 6.8 kg; height: 180.8 ± 6.1 cm; maximal aerobic power: 459.4 ± 31.1 W; VO_2_max: 71.6 ± 4.6 mL/kg/min) 12 female (age: 28 ± 6 yr; body mass: 62.1 ± 8.9 kg; height: 169.1 ± 8.0 cm; maximal aerobic power: 327.1 ± 32.3 W; VO_2_max: 59.9 ± 5.1 mL/kg/min)	TT male: 43.83 km female: 29.35 km	male: ↑ cycling time (p < 0.05) ↑ mean power (p < 0.05) BJ-CAF: 1:02:38.04 ± 0:03:31.00 h*^#^; 314 ± 44 W*^#^ BJ-PLA: 1:04:05.03 ± 0:02:50.09 h; 298 ± 35 W PLA-CAF: 1:02:43.86 ± 0:03:04.87 h*^+^; 313 ± 38 W*^+^ PLA-PLA: 1:03:30.39 ± 0:03:16.15 h; 303 ± 41 Wfemale: ↑ cycling time (p < 0.05) ↑ mean power (p < 0.05) BJ-CAF: 0:51:11.88 ± 0:02:22.13 h*^#^; 212 ± 27 W*^#^ BJ-PLA: 0:51:41.06 ± 0:02:39.51 h; 207 ± 31 W PLA-CAF: 0:50:50.53 ± 0:02:56.48 h*^+^; 216 ± 34 W*^+^ PLA-PLA: 0:51:40.10 ± 0:02:31.71 h; 207 ± 29 W
			140 mL (8.4 mmol ${NO}_{3}^{-}$); 130 min	1			
	CAF		2 mg/kg; 40 min	1			
			1 mg/kg; 10 min	1			
Mero et al. (2013) [42]	BA		4.8 g/day; days before test	28	13 competitive male swimmers (age: 20.5 ± 1.4 yr; body mass: 80.1 ± 8.1 kg; height: 188 ± 8 cm)	2x 100 m TT	↔ swimming time 1 (p > 0.05) ↔ swimming time 2 (p > 0.05) ↑ swimming time difference (p < 0.05)swimming time 1: SB-BA: 57.06 ± 2.97 s SB-PLA: 57.62 ± 2.44 s PLA-BA: 57.14 ± 2.89 s PLA-PLA: 57.06 ± 2.35 s swimming time 2: SB-BA: 58.58 ± 3.22 s SB-PLA: 58.13 ± 2.53 s PLA-BA: 58.55 ± 3.19 s PLA-PLA: 59.03 ± 3.08 s swimming time difference: SB-BA: 1.52 s SB-PLA: 0.51 s* PLA-BA: 1.41 s PLA-PLA: 1.97 s
	SB		0.3 g/g; 60 min	1			
Oskarsson & McGawley (2018) [49]		BJ	70 mL (7.3 mmol ${NO}_{3}^{-}$); 150 min	1	n = 9 healthy endurance runners 7 men (age: 30.4 ± 6.3 yr; body mass: 73.2 ± 8.3 kg) 2 women (age: 31.5 ± 9.2 yr; body mass: 64.2 ± 1.5 kg)	1 km TT	↔ running time (p = 0.540) BJ-CAF: 198 ± 25s BJ-PLA: 200 ± 23s PLA-CAF: 198 ± 24s PLA-PLA: 198 ± 29s
		CAF	4.8 ± 0.4 mg/kg; 45 min	1			
Paton et al. (2022) [55]		BC	300 ml; 40 min	1	12 male amateur cyclists (age: 39.5 ± 11.4 yr; height: 177.9 ± 5.7 cm; weight: 78.2 ± 8.9 kg; and VO_2_max: 4.71 ± 0.61 L/min)	8 x 5 min loads	↑ mean power (p < 0.05) BC-CAF: 311 ± 38 W^#^ BC-PLA: 301 ± 36 W PLA-CAF: 308 ± 37 W^+^ PLA-PLA: 304 ± 38 W
		CAF	4 mg/kg; 40 min	1			
Pruscino et al. (2008) [50]		CAF	6.2 ± 0.3 mg/kg; 120-30 min	1	6 elite male freestyle swimmers	2x 200 m TT	↔ swimming time 1 (p = 0.06) CAF-SB: 2:01.69 ± 3.19 min CAF-PLA: 2:02.42 ± 3.17 min PLA-SB: 2:03.01 ± 3.68 min PLA-PLA: 2:03.77 ± 3.21 min↔swimming time 2 (p = 0.06) CAF-SB: 2:01.70 ± 2.84 min CAF-PLA: 2:03.90 ± 3.58 min PLA-SB: 2:02.62 ± 4.16 min PLA-PLA: 2:04.22 ± 3.75 min
		SB	0.3 g/kg; 45 min	1			
Rezaei et al. (2019) [51]		CAF	6 mg/kg; 50 min	1	n = 8 Karatekas (age: 20.5 ± 2.4 yr; height: 1.78 ± 0.06 m; body mass: 67.8 ± 7.7 kg; body fat: 10 ± 3%)	TTE in a Karate-specific aerobic test	↑ TTE (p < 0.001) CAF-SB: 696 ± 28s* CAF-PLA: 674 ± 44s* PLA-SB: 693 ± 28s* PLA-PLA: 636 ± 39s
		SB	0.3 g/kg/day; divided over the meals on the days before the test	3			
			0.1 g/kg; 120, 90, 60 min	1			
Zart et al. (2024) [52]		BC	600 mg; 120 min	7	n = 2 healthy male recreational cyclists (age: 28.5 ± 4.5 years; body mass: 84.0 ± 9.2 kg)	PWC150 20 min TT	↑ TT-Power (p < 0.05) BC-CAF: 3.37 W/kg (3.30-3.67)* BC-PLA: 3.26 W/kg (3.16-3.47) PLA-CAF: 3.34 W/kg (2.94-3.72)* PLA-PLA: 3.13 W/kg (2.77-3.54)
		CAF	5 mg/kg; 60 min	1			

BA = beta-alanine; BC = blackcurrant; BRC = beetroot crystals; BJ = beetroot juice; CAF = caffeine; CAR = carnitine; IQR = interquartile range; M ± SD = mean ± standard deviation; NEA = nutritional ergogenic aid; PLA = placebo; SB = sodium bicarbonate; TT = time trial; TTE = time-to-exhaustion; VO_2_max = maximal oxygen consumption; ^*^significantly different from placebo; ^+^significant difference between the isolated supplements; ^#^significant difference between combined supplementation and one isolated supplementation

### 3.4. Meta-analysis results

For the meta-analytical analysis, six studies on the supplement combination CAF and SB were identified among the included studies. Because the study by Higgins et al. [53] showed only the median result, it was excluded from the calculations. The combination of CAF and BJ was used in four studies, whereby the study by Lane et al. [57] presented the results for both sexes separately; consequently, the two independent groups were included in the meta-analysis as separate studies.

The average number of participants for the meta-analyses was *n* = 8.8 for the combination CAF with SB and *n* = 12.2 for CAF with BJ. In conjunction with the calculated ES, there was insufficient test power for the meta-analyses (< 51%).

#### 3.4.1. Isolated and combined effects of caffeine and sodium bicarbonate versus placebo

**Figure 4.**
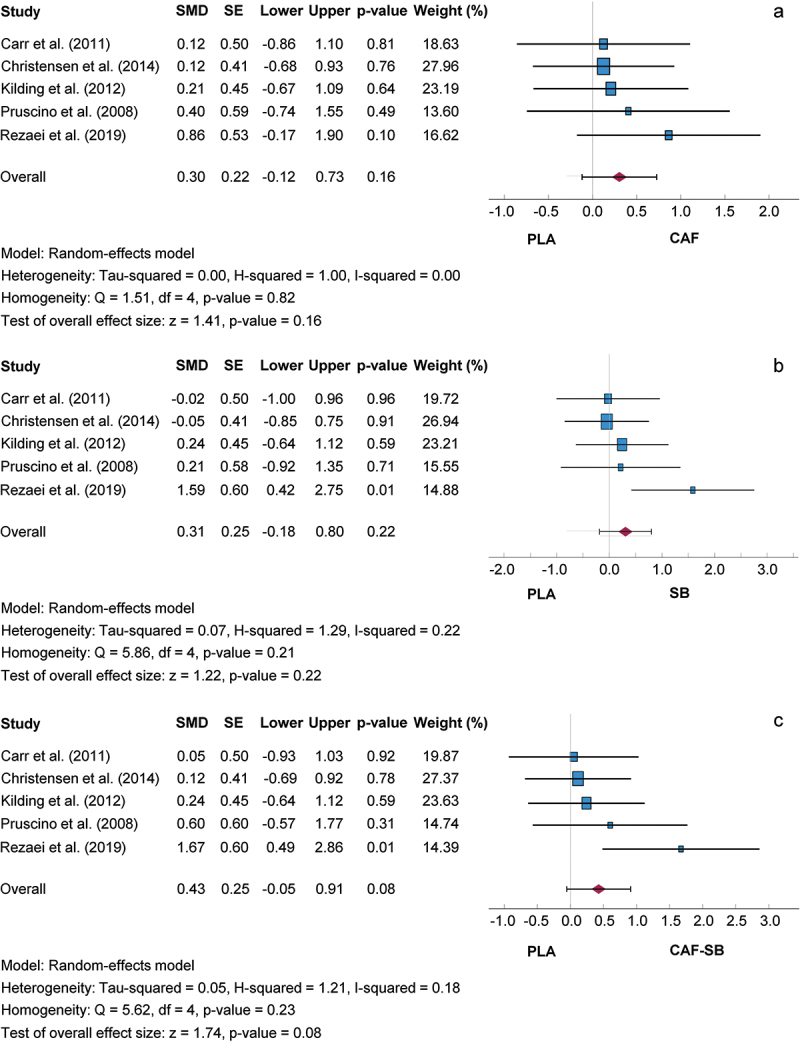
Effect size of caffeine (a), sodium bicarbonate (b) and caffeine + sodium bicarbonate (c) ingestion on exercise performance compared to placebo with 95% confidence intervals. BJ: beetroot juice; CAF: caffeine; SE: standard error; SMD: standardized mean difference.

Figure 4 shows comparisons between the isolated and combined intake of CAF and SB and the PLA trial. The SMD for the CAF/PLA, SB/PLA, and CAF-SB/PLA comparisons were 0.30, 95% CI [−0.12, 0.73]; 0.31, 95% CI [−0.18, 0.80]; and 0.43, 95% CI [−0.05, 0.91], respectively. Consequently, the mean outcome was not significantly different from zero (CAF/PLA: z = 1.41, *p* = 0.160; SB/PLA: z = 1.22, *p* = 0.220; CAF-SB/PLA: z = 1.74, *p* = 0.080). Based on the Q-test, the true outcomes showed no significant heterogeneity in the CAF/PLA (Q (4) = 1.51, *p* = 0.820), SB/PLA (Q (4) = 5.86, *p* = 0.210), or CAF-SB/PLA (Q (4) = 5.62, *p* = 0.230) comparisons. Heterogeneity by I^2^ was very low for the CAF/PLA, SB/PLA, and CAF-SB/PLA comparisons at 0, 22, and 18%, respectively. A sensitivity analysis showed that the study by Rezaei et al [48] caused the heterogeneity without influencing the result. Subgroup analyses carried out did not reveal any additional findings. A 95% prediction interval for the true outcomes ranged from −0.38 to 0.99, from −0.52 to 1.14 and from −0.37 to 1.23 for the CAF/PLA, SB/PLA and CAF-SB/PLA comparisons, respectively, suggesting that both negative and positive true outcomes could occur for the supplements versus PLA despite a positive effect size. The regression test revealed no funnel plot asymmetry for any of the analyses (CAF/PLA, *p* = 0.616; SB/PLA, *p* = 0.247; CAF-SB/PLA, *p* = 0.230).

#### 3.4.2. Isolated and combined effects of caffeine and beetroot juice versus placebo

**Figure 5.**
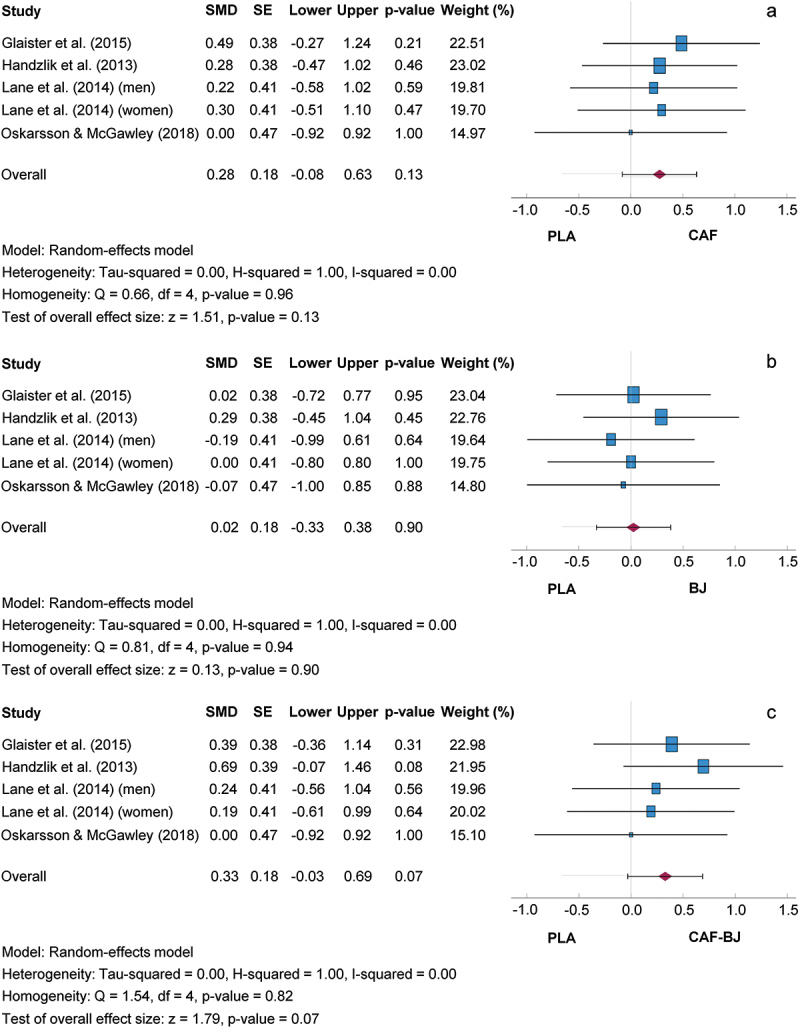
Effect size of caffeine (a), beetroot juice (b) and caffeine + beetroot juice (c) ingestion on exercise performance compared to placebo with 95% confidence intervals. BJ: Beetroot juice; CAF: Caffeine; SE: Standard error; SMD: Standardized mean difference.

Figure 5 shows comparisons between the isolated and combined intake of CAF and BJ and the PLA condition. The SMD for the CAF/PLA, BJ/PLA, and CAF-BJ/PLA comparisons were 0.28, 95% CI [−0.08, 0.63]; 0.02, 95% CI [−0.33, 0.38]; and 0.33, 95% CI [−0.03, 0.69], respectively. Consequently, the mean outcome was not significantly different from zero (CAF/PLA: z = 1.51, *p* = 0.130; BJ/PLA: z = 0.13, *p* = 0.900; CAF-BJ/PLA: z = 1.79, *p* = 0.070). Based on the Q-test, the true outcomes showed no significant heterogeneity in the CAF/PLA (Q (4) = 0.66, *p* = 0.960), BJ/PLA (Q (4) = 0.81, *p* = 0.940), or CAF-BJ/PLA (Q (4) = 1.54, *p* = 0.820) comparisons. The I^2^ statistics showed no heterogeneity for all comparisons. The subgroup analysis with the factor “test protocol” showed an increased SMD of 0.39, 95% CI [−0.00, 0.78] for the comparison of CAF-BJ with PLA when using a bicycle and was just above the significance level (*p* = 0.052). A 95% prediction interval for the true outcomes for the CAF/PLA, BJ/PLA and CAF-BJ/PLA comparisons ranged from −0.31 to 0.86, from −0.55 to 0.60 and from −0.26 to 0.91. In the subgroup analysis, the 95% prediction interval ranged from −0.47 to 1.25. This suggests that both negative and positive true outcomes could occur for supplements versus PLA despite a positive effect size. The regression test revealed no funnel plot asymmetry in any of the analyses (CAF/PLA, *p* = 0.485; BJ/PLA, *p* = 0.665; CAF-BJ/PLA, *p* = 0.366).

#### 3.4.3. Combined effects of caffeine and sodium bicarbonate versus isolated supplementation

**Figure 6.**
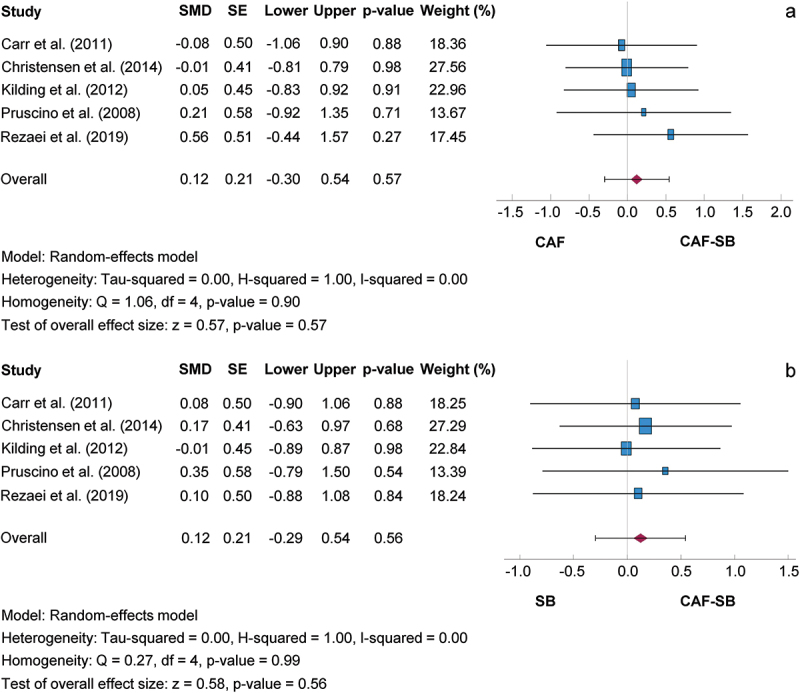
Effect size of caffeine and sodium bicarbonate co-ingestion on exercise performance compared to caffeine (a) and sodium bicarbonate (b) with 95% confidence intervals. BJ: Beetroot juice; CAF: Caffeine; SE: Standard error; SMD: Standardized mean difference.

Figure 6 shows a comparison between the isolated and combined intake of CAF and SB. The SMD for the CAF-SB/CAF and CAF-SB/SB comparisons were 0.12, 95% CI [−0.30, 0.34] and 0.12, 95% CI [−0.29, 0.54], respectively. Consequently, the mean outcomes were not significantly different from zero (CAF-SB/CAF: z = 0.57, *p* = 0.570; CAF-SB/SB: z = 0.58, *p* = 0.560). Based on the Q-test, the true outcomes showed no significant heterogeneity for the CAF-SB/CAF (Q (4) = 1.06, *p* = 0.900) and CAF-SB/SB (Q (4) = 0.27, *p* = 0.990) comparisons. Subgroup analyses carried out did not reveal any additional findings. A 95% prediction interval for the true outcomes for the comparisons CAF-SB/CAF and CAF-SB/SB ranged from −0.56 to 0.80 and from −0.56 to 0.80. This indicates that both negative and positive true outcomes could occur for the supplement combination compared with the isolated intake of supplements, despite a positive effect size. The regression test also showed no funnel plot asymmetry in any of the analyses (CAF-SB/CAF, *p* = 0.694; CAF-SB/SB, *p* = 0.867).

#### 3.4.4. Combined effects of caffeine and beetroot juice versus isolated supplementation

**Figure 7.**
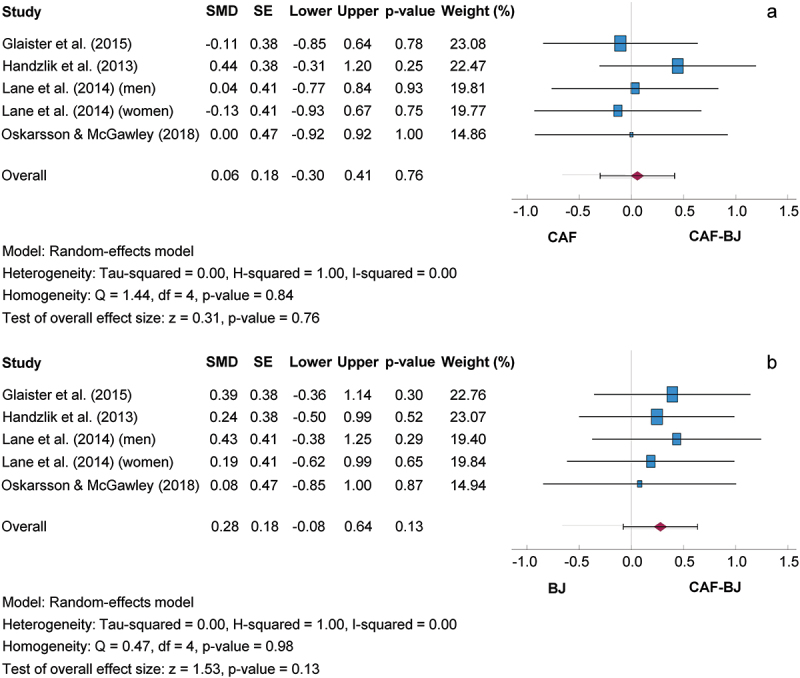
Effect size of combined caffeine and beetroot juice co-ingestion on exercise performance compared to caffeine (a) and beetroot juice (b) with 95% confidence intervals. BJ: Beetroot juice; CAF: Caffeine; SE: Standard error; SMD: Standardized mean difference.

Figure 7 shows the comparison between the isolated and combined use of CAF and BJ. The SMD for the CAF-BJ/CAF and CAF-BJ/BJ comparisons were 0.26, 95% CI [−0.30, 0.41] and 0.28, 95% CI [−0.08, 0.64], respectively. Consequently, the mean outcome was not significantly different from zero (CAF-BJ/CAF: z = 0.31, *p* = 0.760; CAF-BJ/BJ: z = 1.53, *p* = 0.130). Based on the Q-test, the true outcomes showed no significant heterogeneity for the CAF-BJ/CAF (Q (4) = 1.44, *p* = 0.840) and CAF-BJ/BJ (Q (4) = 0.47, *p* = 0.980) comparisons. Subgroup analyses carried out did not reveal any additional findings. A 95% prediction interval for the true outcomes for the CAF-BJ/CAF and CAF-BJ/BJ comparisons ranged from −0.52 to 0.64 and from −0.30 to 0.86. This indicates that both negative and positive true outcomes could occur for the supplement combination compared with the isolated intake of supplements, despite a positive effect size. The regression test showed no funnel plot asymmetry in any of the analyses (CAF-BJ/CAF, *p* = 0.776; CAF-BJ/BJ, *p* = 0.649).

## 4. Discussion

The main purpose of this systematic review and meta-analysis was to analyze and summarize the current evidence regarding the effects of combined supplementation on endurance performance. An overview of the studies included in the review shows that approximately 30% of the significant effects were recorded for the comparisons between an isolated supplement and PLA across all performance parameters. Co-ingestion led to a significant difference in 40% of the outcomes compared with the PLA trial. A significant performance gain through additive compared with an isolated supplement occurred in approximately 17% of the outcomes. The results for the two combinations of supplements analyzed showed that both isolated and combined supplementation had only trivial and small, non-significant effects compared with PLA (all *p* > 0.05). Comparison of co-ingestion with the isolated intake of supplements did not result in any significant improvement in performance (all *p* > 0.05) (Table 4).

**Table 4.** Summary of the results.

Trial	ES	95% CI	95% PI	P-value	Comparable ES from meta-analyses
CAF vs. PLA (CAF-SB-trial)	0.30	−0.12, 0.73	−0.38, 0.99	0.16	0.09–0.49
SB vs. PLA	0.31	−0.18, 0.80	−0.52, 1,14	0.22	0.14–0.94
CAF-SB vs. PLA	0.43	−0.05, 0.91	−0.37, 1.23	0.08	
CAF vs. PLA (CAF-BJ-trial)	0.28	−0.08, 0.63	−0.31, 0.86	0.13	0.09–0.49
BJ vs. PLA	0.02	−0.33, 0.38	−0.55, 0.60	0.90	0.09–0.79
CAF-BJ vs. PLA	0.33	−0.03, 0.69	−0.26, 0.91	0.07	
CAF-SB vs. CAF	0.12	−0.30, 0.34	−0.56, 0.80	0.57	
CAF-SB vs. SB	0.12	−0.29, 0.54	−0.56, 0.80	0.56	
CAF-BJ vs. CAF	0.26	−0.30, 0.41	−0.52, 0.64	0.76	
CAF-BJ. Vs. BJ	0.28	−0.08, 0.64	−0.30, 0.86	0.13	

BJ = Beet root juice; CI = Confidence interval; CAF = Caffeine; ES = Effect size; PI = Prediction interval; PLA = Placebo; SB = Sodium bicarbonate.

Combined CAF and SB appeared to complement each other in terms of percentage performance development. Comparison of the isolated supplements with those of the PLA trial showed a small increase in performance (CAF-PLA, 0.88%; SB-PLA, 0.36%). When the two supplements were combined, the performance further improved (CAF/SB-PLA: 0.95%). Co-ingestion led to a trivial effect (CAF-SB/PLA: ES = 0.43, *p* = 0.08) compared to isolated supplements (CAF/PLA: ES = 0.30, *p* = 0.16; SB/PLA, ES = 0.31, *p* = 0.22). Specifically, adding CAF to SB (0.60%) resulted in a greater performance increase than adding SB to CAF (0.08%). In principle, all integrated trials in which CAF was administered as a supplement showed more significant improvements than the PLA, SB, and BJ trials, even though these performance increases were below the 2–3% reported in other studies [3,19]. The increase in performance through a combination of CAF and SB could have been caused by the suppression of fatigue symptoms. CAF attenuates the fatiguing effect of adenosine by blocking adenosine receptors and could therefore reduce the perceived exertion [17]. SB also ensures that an increase in blood bicarbonate improves the efflux of hydrogen ions and lactate from the working muscles and counteracts intramuscular acidification and consequently fatigue [17]. The described effects of the supplements on the body are independent of training status and have been confirmed in both elite athletes and recreational athletes [24,58]. Furthermore, it was even shown that in elite athletes with a VO_2_max >75 ml/kg/min CAF was able to improve VO_2_max by 1.2% by increasing maximum heart rate and maximum ventilation [59].

The performance-enhancing potential of CAF has been demonstrated in trials with BJ (CAF/PLA: ES = 0.28, *p* = 0.13; CAF-BJ/PLA: ES = 0.33, *p* = 0.07). On average, a maximum increase in performance of 1.45% was observed in four out of five trials in which a time trial was conducted with CAF and BJ supplements. Improvement in performance was observed when comparing the co-ingestion of CAF-BJ and BJ (ES = 0.28, *p* = 0.13). CAF increased performance (1.22%) and BJ decreased performance (−0.46%) compared with PLA. Similarly, when comparing the isolated and combined effects of the CAF and BJ supplements, the results showed a deterioration in co-ingestion when CAF-BJ was compared with CAF (−0.23%) and an improvement with the combination when CAF-BJ was compared with BJ (1.45%). Consequently, it can be assumed that the ergogenic effect of CAF was not further supported by BJ during endurance exercise, although the nitrate contained in BJ is converted to nitric oxide (NO) in the body and is supposed to ensure better blood flow and increased muscle contraction [49]. In contrast to performance in time trials is the result of the study by Handzlik et al. [41]. After a preload at 60% VO_2_max over 30 min, the subjects with BJ in the TTE showed an increase of 23% at 80% VO_2_max compared with the placebo condition, and thus showed a greater increase in performance than in the CAF trial (15%). The combination of both supplements led to a further substantial but non-significant increase of 46%. However, the result was not significant only because there was a very high inter-individual variability in the results. Nevertheless, the study indicates the combined performance-enhancing potential of the supplements [41].

Significant performance-enhancing effects were found for beetroot (SMD = 0.15) during exercise lasting up to 10 min [6]. Four of the five studies included in the meta-analysis [40,41,57] did not fall within this time window, and therefore could suppress the small effect observed with BJ. Furthermore, no effects of nitrate supplementation reported in male subjects with a VO_2_max >65 ml/kg/min [6]. In the meta-analysis by van de Walle et al. [23], an effect of BJ was only demonstrated in untrained subjects, regardless of sex. The trained subjects did not experience any increase in performance through BJ. Since the female and male subjects in three studies were professional road cyclists [40,57] and in the study by Handzlik et al. [41] the VO_2_max of 63 ± 10 mL/kg/min was just below the established ineffective performance threshold, the performance level of the subjects could also have had an effect on the results. Another possibility would be that the test protocols in the studies caused a high utilization of VO_2_max and thus BJ could no longer exert its vasodilatory effect because vasodilatation, blood distribution and cardiac output were already close to the maximum. According to Larsen et al. [60], oxygen costs were reduced during submaximal exercise, but this effect no longer occurred during exercise ≥ 80% VO_2_max. One reason for this observation above a load of 80% VO_2_max could be the dominance of anaerobic metabolic pathways to cover the rapid energy demand. The NO-mediated efficiency improvement in mitochondrial respiration and ATP consumption decreases. Consequently, the aforementioned studies could not identify any differences between the BJ and PLA trials.

Looking at the change in performance in relation to the variability coefficient of some ergometric tests (e.g. rowing, cycling), it can be stated that in all studies the assumed variability of 0.6 to 2.7% in the time measurement already exceeded the observed increase in performance. This means that there is no practically relevant increase in performance, as it cannot be clarified whether an increase in performance of 1% is merely suggested by the measurement variability or whether there is really an improvement [61].

As the number of studies investigating the isolated and combined effects of supplements in a blinded and randomized crossover design was manageable and few studies were available for a specific supplement combination, the significance of this systematic review and meta-analysis is limited. Regarding the results of the primary studies of the systematic review, it was found that in only five studies, co-ingestion led to a significant difference compared to one of the trials with isolated supplementation [38,53,55–57]. Finally, the pooled results of the meta-analyses also did not confirm the existing findings on ergogenic effects when using the supplements CAF, SB and BJ in isolation. An additional benefit of combining CAF with SB and BJ was not evident in the aggregate results. However, the evidence is inconclusive due to the limited number of studies and further studies are needed to clarify the ergogenic effect of supplement combinations.

With regard to the design of the studies, it can be summarized that the primary studies in their designs administered supplements at a sufficient minimum dose with the correct time interval to the tests [22,28,62]. In addition, a homogeneous group of elite and sub-elite athletes was examined, which should lead to greater conclusiveness. There were no differences in the dosage of the supplements in SB (0.3 g/kg). The amount of caffeine, on the other hand, was between 3 and 6 m/kg in the studies, but a substantial increase in performance due to a higher dose cannot be deduced from the effect sizes. The same can be said for the BJ trial. Although the studies have followed general recommendations for the intake of supplements to achieve ergogenic effects, there are moderators that can explain the heterogeneous outcomes. In particular, studies on caffeine show that the change in performance varies between individuals [63]. The differences may be due to a person’s CYP1A2 genotype and the resulting caffeine metabolism [64]. In addition, habituation to caffeine can occur with daily intake, so that a dose that does not sufficiently exceed the habitual dose could not trigger ergogenic effects [65,66]. The choice of test can also determine whether an ergogenic or ergolytic effect is recorded in a participant [67]. And it has been shown that the ergogenic effect is enhanced in trained athletes [66]. The opposite is true for BJ, which had a reduced effect with increasing training status [6]. In the primary studies, the intake of SB was uniformly set between 45 and 150 min before the test. Individual time-to-peak protocols were not considered, so it could not be guaranteed that each participant completed their test at the optimal time [68]. Fundamental problems with supplement studies are blinding and the placebo and nocebo effect. Despite a careful blinding procedure for the participants and the investigators, supplements can cause side effects (e.g. nausea, headaches), so that blinding is only possible to a limited extent. Therefore, the absence of a control of blinding is a limitation of a study and this case occurred fourteen times. In addition, the participants’ belief that a supplement causes an ergogenic effect or fails to do so can trigger a placebo effect or a nocebo effect [69,70]. In studies with nutritional ergogenic aids, large placebo effects (d = 0.86) were found on the measurement [71]. Conflicts of interest as a further problem could be excluded in 10 of 16 studies. The remaining studies did not provide any information on this.

The pooled effect sizes were always trivial or small and the corresponding confidence intervals only provided an imprecise estimate of the mean effect due to their width. Compared to other studies on the isolated effect of supplements, it can be noted that most of the effect sizes from meta-analyses with CAF, BJ and SB were also small at most (0.14 < ES < 0.49) [13,22]. When the studies analyzed only TTE protocols, moderate and large effect sizes of 0.79, 95% CI [0.23, 1.35] [21] and 0.94, 95% CI [0.00, 2.87] [26] were reported. Thus, the trials with CAF (ES = 28 and 30) and SB (ES = 0.31) confirm the previous results. In contrast, our study does not even begin to detect a proven increase in performance with BJ (ES = 0.02). A major difference to our study is that considerably more primary studies could be included in the meta-analyses on the isolated effect. In practice, it can be concluded that although the effect sizes of CAF and SB could have a small improvement on competition performance, no clear direction for the effect of CAF and SB can be determined due to the 95% prediction intervals. The results of this systematic review with meta-analysis are based on small studies with populations ranging from elite to sub-elite athletes. The available data do not allow a clear statement to be made about the effectiveness of a supplement or a supplement combination, as BJ, for example, has been shown to have no effect above a certain performance level. Unfortunately, due to the small number of studies with similar populations, it has not been possible to draw any clear conclusions. Due to the low power of the meta-analytical results, the statistical likelihood that a significant result would reflect a true effect is reduced. In addition, the more important aspect for this study is that the chance of detecting a true effect is also lower. In some cases, despite contacting the authors, the data had to be extracted from the figures using the WebPlot Digitizer tool. Even if this tool can be used reliably, there may still have been inaccuracies when extracting the values, which could have influenced the results of the meta-analyses. A strength of this meta-analysis is that no restrictions were placed on the selection of ergogenic supplements. All possible combinations were considered for the review. Consequently, the reader can easily see that the research findings are limited and only very few combinations were examined in several studies. Further studies are therefore needed in the future to be able to make a solid statement as to whether a co-ingestion of two supplements could achieve a synergistic effect.

## 5. Conclusion

Current evidence suggests that co-ingestion of two supplements does not consistently produce a significant performance benefit. However, further studies with standardized methodologies are needed to confirm this finding.

Based on the larger number of meta-analyses that have demonstrated an ergogenic effect for the substances CAF, SB and nitrates, it seems advisable to use a co-ingestion of these supplements in future studies, particularly as the physiological effects of the supplements could support each other with regard to the reduction of fatigue symptoms. The studies should be conducted as double-blind, randomized controlled crossover studies with endurance-trained athletes and investigate the four conditions placebo, supplement 1, supplement 2 and supplement 1 + 2 using a reliable and standardized test protocol (e.g. cycling time trials in the laboratory). The ergogenic doses for CAF (3–6 mg/kg), SB (0.3 g/kg) and BJ concentrate (140 ml/day) should be considered, whereby CAF and SB should be taken shortly before exercise (60 and 60–120 min respectively) and BJ optionally only in advance on the day of exercise (120–210 min) or additionally over 3–5 days before the test.
